# Exploring Gender Disparities in Emotional Intelligence, Leadership Access, and Career Development Among Jordanian Nurses

**DOI:** 10.1155/jonm/8824621

**Published:** 2025-09-08

**Authors:** Wesam Taher Almagharbeh, Hazem AbdulKareem Alfanash, Khaldoon Aied Alnawafleh, Amal Ali Alasmari, Amal Ali Alharbi, Malik A. Altayar, Sameer A. Alkubati, Wafa Hamad Almegewly, Khulud Ahmad Rezq, Elham H. Othman

**Affiliations:** ^1^Medical Surgical Nursing Department, Faculty of Nursing, University of Tabuk, Tabuk, Saudi Arabia; ^2^Department of Nursing Leadership and Education, Faculty of Nursing, University of Tabuk, Tabuk, Saudi Arabia; ^3^Department of Medical Laboratory Technology, Faculty of Applied Medical Sciences, University of Tabuk, Tabuk, Saudi Arabia; ^4^Department of Medical Surgical Nursing, College of Nursing, University of Ha'il, Ha'il, Saudi Arabia; ^5^Department of Community and Psychiatric Mental Health Nursing, College of Nursing, Princess Nourah bint Abdulrahman University, P.O. Box 84428, Riyadh 11671, Saudi Arabia; ^6^Community & Psychiatric Health Nursing Department, Faculty of Nursing, University of Tabuk, Tabuk, Saudi Arabia; ^7^Faculty of Nursing, Applied Science Private University, Amman, Jordan

**Keywords:** career development, emotional intelligence, gender disparities, healthcare workforce, Jordanian healthcare, nursing, professional development

## Abstract

**Purpose:** This study explored gender differences in emotional intelligence (EI), leadership access, career development, and professional development among Jordanian nurses (*n* = 186), aiming to identify institutional barriers affecting female nurses' leadership trajectories.

**Methods:** A quantitative cross-sectional study was conducted across public, private, and military hospitals in Jordan using convenience sampling. Data were collected using validated scales for EI, career development, leadership access, and professional development. Statistical analyses included independent samples *t*-tests and multiple regression, with ethical approval obtained from the relevant institutional review boards.

**Findings:** Female nurses scored significantly higher in EI (*M* = 84.2, SD = 4.7) than males (*M* = 78.5, SD = 5.1, *t* = −3.45, *p*=0.001). However, males reported greater leadership access (*M* = 6.2, SD = 1.8) than females (*M* = 5.4, SD = 2.1, *p*=0.034). Regression analysis showed that higher EI predicted leadership access (*β* = 0.450, *p*=0.001), yet gender remained a significant negative predictor for females (*β* = −0.410, *p*=0.030), indicating institutional barriers rather than an EI deficit. No significant gender differences were found in career development (*p*=0.442), and professional development scores showed only minor differences between genders without statistical significance.

**Discussion:** Despite having higher EI, female nurses face structural challenges in accessing leadership positions. These findings underscore the need for gender-inclusive leadership pathways and organizational reforms that address systemic inequities.

## 1. Introduction

The quality and service delivery in Jordan's healthcare sector have improved significantly in recent years, particularly in areas such as patient satisfaction, workforce expansion, and institutional accreditation. However, despite these improvements, gender disparities remain prominent, particularly in emotional intelligence (EI), career development (CD), leadership access (LA), and professional development (PD) [1]. According to the Jordanian Nurses and Midwives Council (JNMC) [2], in Jordan, 67% of the nursing workforce is women, but only 20% of them hold leadership roles.

While women outnumber men in nursing, structural barriers continue to limit their advancement into senior management positions. These barriers include unequal access to promotion pathways, leadership training, and decision-making roles, reflecting systemic rather than individual limitations. The gender disparities observed in Jordan mirror global trends and warrant an in-depth, context-specific investigation into nursing leadership imbalances.

Gender inequity in nursing leadership is a documented global phenomenon. Despite being the numerical majority, women nurses in countries such as the United States, the United Kingdom, and Australia continue to experience stagnant representation in leadership roles [3, 4]. In Middle Eastern and developing countries, cultural and institutional constraints further exacerbate this underrepresentation, compounding workplace inequality. Similar patterns have been observed in the Gulf region and North Africa, as reported by Pincha Baduge et al. [5].

This study contributes to the global discussion on gender equity in nursing leadership by focusing specifically on the Jordanian healthcare sector. It aims to provide empirical evidence that can inform policy development and workforce strategies to mitigate gender-based barriers to leadership.

To ensure conceptual clarity, this study focuses on four interrelated dimensions as follows:

• EI: It is the ability to understand, manage, and regulate emotions to enhance decision-making and social interaction [6, 7]. Although higher EI is linked with leadership capability, gender-based barriers may diminish its effect on career progression for female nurses [8].
• CD: CD typically includes education, mentorship, and promotion opportunities [9]. It is essential for specialization and leadership readiness, but many female nurses encounter institutional and societal limitations that delay their advancement.
• LA: It is the extent to which individuals can attain managerial roles through training, promotion structures, and organizational policy [10]. Men in Jordan are more frequently offered leadership training, giving them an advantage in career progression [11].
• PD: It refers to specialized training, workshops, and continuing education [12]. Cultural restrictions and logistical barriers, such as evening sessions or travel requirements, further limit women's participation in PD [13].

## 2. Background

Gender barriers in nursing are not only individual-level issues but are embedded within organizational systems [14]. Studies confirm that women are less frequently selected for leadership training, often a prerequisite for career progression [15].

Female nurses often experience cumulative disadvantage, as multiple systems simultaneously limit access to leadership training, mentorship, and decision-making roles [16–18]. Although EI is a strength among many female nurses [19], this advantage does not always translate to leadership promotion due to structural constraints [8].

The role of PD in reducing burnout and increasing job satisfaction has been highlighted [12, 20], yet logistical challenges still disproportionately affect women [13]. Lorber and Dobnik [21] also emphasized the role of continued training in building resilience during healthcare crises, but the link to gender disparity remains underdeveloped unless clearly contextualized.

These variables, EI, CD, LA, and PD, do not operate in isolation. Their intersections create complex pathways that may either support or inhibit leadership advancement, especially for women.

This study aims to explore gender disparities in EI, LA, CD, and PD among nurses in Jordanian healthcare settings.

Specifically, the objectives are to

1. Assess the extent of gender disparities in EI, LA, CD, and PD among Jordanian nurses.
2. Evaluate the relationship between EI and LA across gender groups.
3. Analyze the structural and institutional barriers contributing to gender-based inequalities in nursing leadership.

The LA Scale (LAS) (Appendix B), developed and validated for this study, offers a novel tool to measure nurses' perceptions of institutional support for leadership pathways. This tool is introduced in the methodology section but is flagged here to signal its unique contribution.

### 2.1. EI

EI plays a crucial role in leadership and team management, especially in healthcare. Prezerakos [8] reviewed several studies on the EI of nurse managers and found that higher levels of self-directed and other-directed EI were significantly associated with improved leadership performance. EI nurse managers demonstrated better stress management, decision-making, and interpersonal communication skills, all vital for effective healthcare team management. In addition, Abraham and Scaria [22] highlighted that EI not only enhances leadership but also contributes to the overall success of nursing practice. They found that higher EI fosters empathy, unity, and teamwork among staff, leading to improved patient care and interstaff cooperation. This aligns with Ohlson and Anderson's [23] study, which showed that nurse managers with higher self-rated EI were more effective in influencing staff, boosting staff satisfaction, and, consequently, improving patient satisfaction. Also, EI is not only associated with leadership potential but also with reflective thinking skills vital for clinical decision-making and professional growth [24].

Al-Oweidat et al. [19] solely targeted the link between EI and organizational commitment with nurses in Jordan. In their work, they found that EI was positively related to organizational commitment and negatively related to turnover intention. This implied that EI could be a useful variable for leadership and also for the overall nurse retention and organizational stability.

Altogether, these studies placed EI at the center of the nursing leadership agenda. Studies that compared high and low EI nurses indicated that the former were more effective in team management, creation of a healthy work environment, and quality patient care. The need for EI was also highlighted in terms of leadership opportunity and promotions, because those nurses who possess such a competency are likely to be promoted for leadership roles.

### 2.2. CD

Promotion of nurses to leadership roles was the main area of career advancement. Gunawan and Aungsuroch [9] identified the key skills and qualities of first-line nurse managers and determined that career advancement programs such as education, mentorship, and leadership development were vital for career advancement. They further asserted that the nurses who engaged in CD plans and training had enhanced managerial and leadership competencies that are relevant at a higher tier (it refers to senior leadership roles or higher levels of professional responsibility).

Sfantou et al. [25] performed a systematic review to identify the effects of leadership styles and CD programs on the quality of care in healthcare organizations. Their review identified that those nurses who have had access to well-defined structured career advancement programs had an increased likelihood of assuming leadership roles and enhancing patient care. The relationship between PD and leadership indicated that the funding for nurse education and training was associated with the enhancement of healthcare quality.

Vyas [26] studied the concept of EI in career advancement and concluded that the leadership training that improves EI has played an influential role in the nurses' leadership. She said this was because the application of EI training in CD programs would produce better leadership results. It was identified that nurses who were trained to regulate their emotions and build cooperation skills were rated as better leaders, as well as reported better patient and staff results.

The literature pointed out that career advancement was an important determinant of leadership opportunities in nursing. Structured courses, apprenticeship, and leadership training programs enabled the nurses to gain the right skills needed for them to advance to the management level positions. However, the studies also revealed that not all the nurses could get these opportunities as often as they wanted, as this depended on the policies of their institution and the availability of resources.

### 2.3. LA

LA in the nursing profession continued to be a problem especially with regard to gender. Babalola [15] examined the relationship between LA, organizational commitment, leadership style, job satisfaction, and employee–supervisor relationship. His research concluded that gendered organizational practices in nursing organizations defined leadership opportunities and individual attributes, including job performance and relational skills, which formed an important part of the leadership promotion criteria.

Nielsen et al. [10], using bibliometric data to examine the distribution of gender in leadership positions in the medical research literature, found that although the healthcare professions are mainly populated by women, men have much better access to leadership positions. They pointed out that there were issues of systematic prejudice on this at the corporate and societal structures that explained this difference. Jacobs et al. [27] observed that women are still locked out from leadership roles in both the medical and nursing professions, even if they possess the required education and experience.

Deng et al. [11] investigated the gender differences in self and others' EI and LA among the nursing students. They were able to establish that male students were promoted more to leadership positions even when they had lower EI than female students. This was made to draw attention to the fact that gender stereotyping pervaded the leadership opportunities available for the male-dominated nursing profession, where male nurses were considered more capable of leadership positions regardless of their actual abilities.

Altogether, these studies showed that LA was not only the merit or essential skill and qualification but also a function of organizational structures and gender discrimination. EI and professional competency were critical as antecedents of leadership, but were insufficient to ensure leadership in nursing, especially for women.

### 2.4. PD Opportunities

Nurses participating in PD programs emphasized that access to training and career advancement opportunities is crucial for career growth. Saygili et al. [12] investigated the correlation of a high work–life quality (W-LQ), burnout, and PD in healthcare workers (HCWs) employed in Turkey. In their study, they highlighted that the nurses who undertook the PD programs had low burnout levels and high job satisfaction. It showed that the nursing profession ought to be a learning and CD field for health and CD.

Zaghini et al. [13] explored the relationship between attending PD workshops and job satisfaction and work–life balance in Italian cardiovascular nurses. They showed from their research how PD resources helped nurses reduce stress, improve their proficiency, and put them in a better place to take on leadership roles. It also helped to validate that education and training were important throughout the working life as well as for general health.

The purpose of this study was to investigate the effect of coronavirus disease 2019 (COVID-19) on the quality of work–life and career advancement [21]. Lorber and Dobnik [21] found that nurses who were able to continue with their continuing PD (CPD) throughout the pandemic crisis were in a better position to meet the challenges that the pandemic crisis introduced. According to their study, there was a need to ensure that nurses are allowed to get training and development especially at a time when the healthcare environment is transforming at a fast pace.

EI plays an important role in leadership success in a professional setting, as it has an influence on decision-making, communication, and other's relations [28]. Women appear to possess higher EI than men in the context of nursing leadership research, yet this EI advantage does not always translate into women's leadership opportunities and may suggest structural barriers to individual competencies and nursing leadership disparities [29]. Variables of education, experience, professional skills, or work hours have influenced the career progression. However, EI is especially important for nursing, as it directly responds to the care for the patients and the management of the team [30]. However, gendered organizational structures often hinder women's career advancement despite their emotional competencies (essential skills and qualifications). This study focuses on EI, CD, LA, and PD as key variables, as they collectively shape leadership trajectories in nursing, where gender disparities remain pronounced [31].

Although previous research highlighted gender disparities in LA, it often fails to distinguish between individual limitations and systemic obstacles. This study moves beyond descriptive accounts of inequality by analyzing how institutional policies and PD opportunities mediate gender differences in leadership attainment [4]. The literature review integrates findings on EI, career progression, and LA, illustrating how these variables interact within organizational frameworks. Rather than presenting isolated studies, this section synthesizes existing research to demonstrate how gendered workplace dynamics limit career mobility for female nurses, emphasizing the need for policy interventions that foster equitable leadership pathways.

## 3. Methods

Describing the methodological approach of this work, this section aims to explore gender disparities in education, access to leadership positions, and career advancement of nurses in the healthcare sector of Jordan. A quantitative research approach was used to support the research findings, which also helped in igniting the external credibility of the work, including reliability and validity. The methodology section outlines the proposed research approach, the way the sample will be selected, the data collection tool, statistical analysis that will be applied, and the issue of ethics [25, 32].

### 3.1. Research Design

This study employed a quantitative cross-sectional design to investigate gender disparities in EI, CD, LA, and PD among Jordanian nurses. This design was selected to assess relationships and differences between variables at a single point in time. The structured format of the research allowed for hypothesis testing and examination of associations between gender and key predictors of LA in nursing.

### 3.2. Settings and Sampling

Hospitals were purposively selected to represent structural and organizational variation across Jordan's public, private, and military healthcare sectors. Hospital size was defined by the number of employed full-time nurses. Within each facility, convenience sampling was used to recruit participants due to logistical constraints and accessibility. This sampling strategy allowed broad participation across departments and roles while acknowledging practical limitations.

The final sample included 186 full-time nurses, both male and female, across leadership and staff positions. Eligibility criteria included having at least 2 years of nursing experience. Nurses on leave, part-time, or with less than 2 years of service were excluded to ensure comparability and professional maturity. Managers and nonmanagers were both included to reflect variation in leadership exposure. The target sample size was calculated based on power analysis using SPSS, indicating that a minimum of 180 participants would be needed to detect medium effect sizes with 80% power at *α* = 0.05.

### 3.3. Measurement Instruments

Four main instruments were used. The EI Scale (EIS), consisting of 33 items, was translated from English to Arabic using forward-backward translation. To ensure cultural and conceptual equivalence, the translated version was reviewed by a panel of bilingual nursing faculty to confirm item clarity and contextual appropriateness.

The LAS, developed for this study, consists of 10 items on a 5-point Likert scale (1 = strongly disagree to 5 = strongly agree). Items assessed mentorship availability, training access, promotion fairness, and perceived encouragement to assume leadership roles (Appendix C). Content validity was established through expert review. Principal component analysis confirmed unidimensionality, with one dominant factor explaining 46.8% of variance and all item loadings exceeding 0.60. Internal consistency was high (Cronbach's alpha = 0.85).

The CD Scale (CDS) included 6 items focused on access to mentorship, promotion tracks, and skill-building opportunities. The PD subscale consisted of 5 items on continuing education, workshops, and skill enhancement. Both scales demonstrated strong internal consistency (CDS *α* = 0.83; PD *α* = 0.81). Item counts were based on content mapping against relevant constructs in the nursing literature and confirmed by expert consensus.

To address language or literacy challenges, trained facilitators were available to provide clarification during survey completion, though all participants demonstrated adequate literacy and completed the questionnaire independently. The questionnaire was self-administered during work breaks and returned in sealed envelopes to preserve anonymity.

### 3.4. Pilot Testing and Validation Process

A pilot study involving 30 nurses across all three hospital sectors was conducted prior to the main study. The goal was to evaluate item clarity, cultural fit, and internal consistency. The pilot sample did not overlap with the main sample. Feedback from pilot participants and experts informed minor revisions to item phrasing. Cronbach's alpha values for all scales exceeded 0.80, supporting internal reliability for the full sample.

### 3.5. Cronbach's Alpha and Reliability Scores

The CDS included 6 items focused on access to mentorship, promotion tracks, and skill-building opportunities. The PD subscale consisted of 5 items on continuing education, workshops, and skill enhancement. Both scales demonstrated strong internal consistency. Item counts were based on content mapping against relevant constructs in the nursing literature and confirmed by expert consensus.  Table 1 presents the internal consistency statistics for each instrument.

### 3.6. Data Collection Procedure

Data were collected from May to July 2024. Institutional Review Board (IRB) approval was obtained from participating hospitals. Surveys were distributed physically during nurse shifts and collected after 2 weeks. All responses were anonymous, and informed consent was obtained from all participants. The Arabic version of the questionnaire was used uniformly.

A total of 200 questionnaires were distributed, with 186 completed and valid responses included in the analysis. Incomplete forms (> 20% missing data) were excluded. Proportional distribution ensured representation across facility type and nurse role.

### 3.7. Statistical Analysis

Data were analyzed using IBM SPSS Statistics Version 25. Descriptive statistics were used to summarize participant characteristics and scale scores. Normality was assessed using the Kolmogorov–Smirnov and Shapiro–Wilk tests. EI, LA, and CD scores were compared across gender using independent samples *t*-tests. Bonferroni correction was applied for multiple comparisons.

Regression analysis was used to examine predictors of LA. Variables included gender, EI, age, education, current position, and years of experience. CD was excluded from the regression model due to conceptual and statistical overlap with LA. Both variables represent structural access to advancement pathways, including mentorship, training, and promotions, which can lead to collinearity and inflated estimates. Variance inflation factors (VIFs) confirmed acceptable multicollinearity levels for the remaining predictors (all VIF < 1.51).

Missing data were handled using pairwise deletion. This method maximized usable data for each analysis but may limit comparability across regression models. This limitation is acknowledged in the discussion.

### 3.8. Ethical Considerations

This study was conducted in accordance with the ethical principles outlined in the Declaration of Helsinki [33] and with the ethical standards of the IRBs. Ethical approval was obtained from the following institutions: Al-Bashir Hospital Institutional Review Board (Approval Number: MOH IRB/2024/181); the Royal Jordanian Medical Services Institutional Review Board (Approval Number: RJMS IRB/2024/073); and the Institutional Review Board of Jordan University Hospital (Approval Number: JUH IRB/2024/NURS/501). Informed consent was obtained from each participant, who was assured that their participation was voluntary and that they could withdraw at any time without consequence. Survey data were anonymized and stored in a secure, password-protected database. Hard copies were stored in locked cabinets and will be destroyed 5 years poststudy in accordance with institutional guidelines.

## 4. Results

This section presents descriptive statistics and comparative analyses exploring gender-based differences in EI, LA, and PD among nurses in Jordan.

### 4.1. Descriptive Statistics

#### 4.1.1. Demographic and Professional Characteristics (*N* = 186)

The sample was selected to include nurses from public, private, and military hospitals to ensure representation across different healthcare environments (Table 2).

#### 4.1.2. EI Levels (*N* = 186)

Table 3 categorizes the EI levels of the participants. EI is a critical factor in nursing leadership, and these data provide insights into how nurses perceive and apply EI in their professional roles.

**Figure 1:**
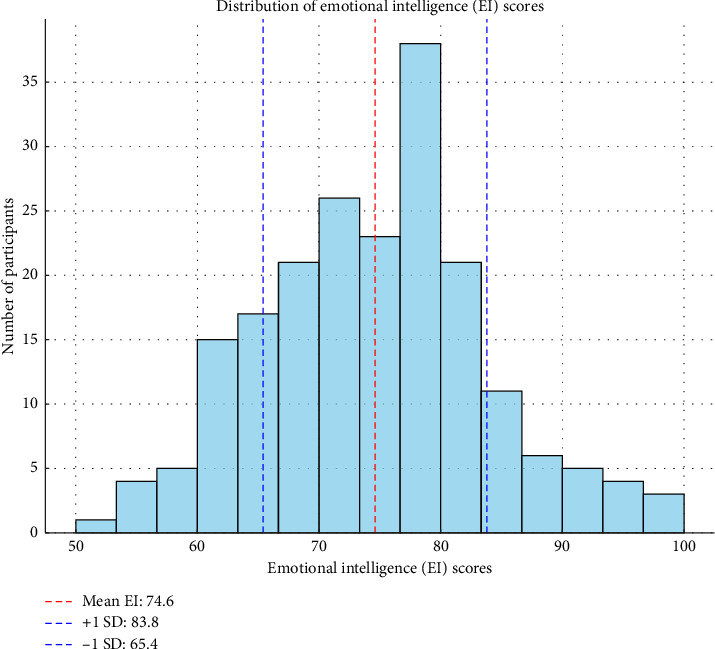
Distribution of emotional intelligence levels (*N* = 186).

This histogram presents the distribution of EI scores among participants, treating EI as a continuous variable rather than predefined categories. The red dashed line (mean EI: 74.8) indicates the average score, while the blue dashed lines (+1 SD: 84.2; −1 SD: 65.4) mark the standard deviation range, highlighting where most scores fall. The bell-shaped distribution suggests approximate normality, supporting statistical analyses that assume normality (Figure 1).

Table 4 presents the descriptive statistics for key variables, including age, years of experience, EI, LA, CD opportunities, leadership training programs attended, and PD workshops. These statistics provide insights into the general characteristics and experiences of male and female nurses in the sample.

**Figure 2:**
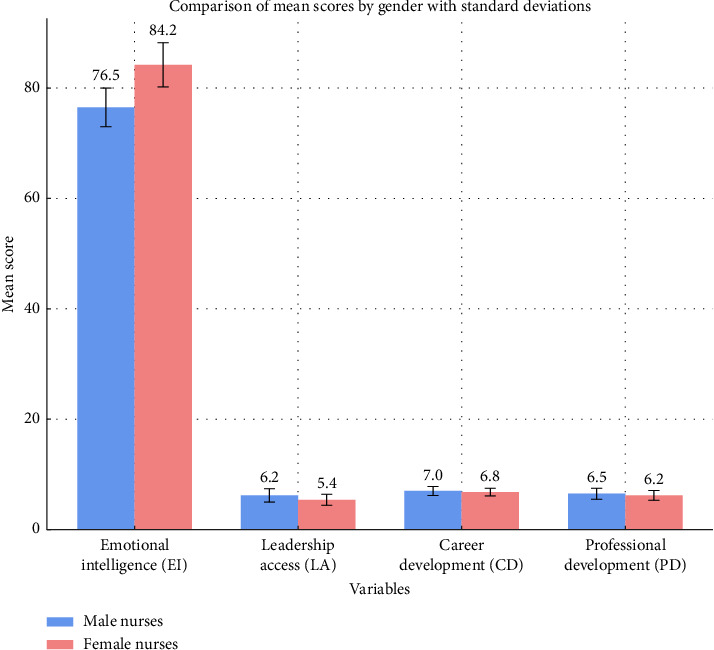
Comparison of key variables between male and female nurses.

This bar graph compares mean scores of EI, LA, CD, and PD between male and female nurses, incorporating standard deviation error bars for accuracy (Figure 2).

### 4.2. T-Test Analysis

#### 4.2.1. *T*-Test Analysis of EI, LA, and CD

This section presents the results of the independent samples *t*-test that was conducted to compare the means of male and female nurses across three key variables: EI, LA, and CD opportunities. The *t*-test was used to assess whether there were significant differences between male and female nurses in these areas.

#### 4.2.2. *T*-Test Results

Table 5 summarizes the results of the *t*-test, including the means for each group, *t-*values, and *p* values.

**Figure 3:**
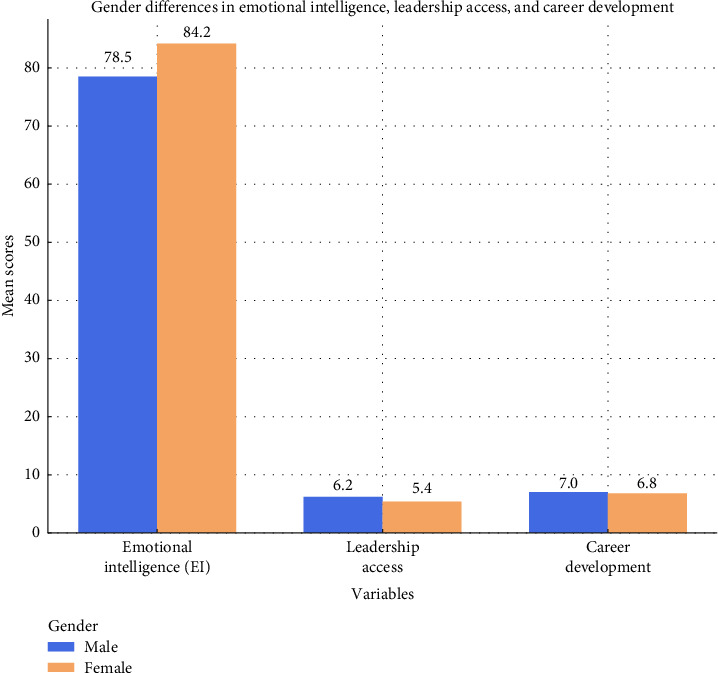
Gender differences in emotional intelligence, leadership access, and career development.

Figure 3 also shows the percentage differences in EI, LA, and CD between male and female nurses, single data points, and confidence intervals.

### 4.3. Regression Analysis

Regression analyses were conducted to identify predictors of LA among nurses, controlling for age, educational level, and years of experience. As shown in Table 6, EI was a significant positive predictor (*β* = 0.45, *p* < 0.001), indicating that higher EI scores are associated with greater perceived LA. Gender was also a significant predictor (*β* = −0.41, *p*=0.03), with male nurses reporting higher LA compared to females. Other variables, including age, education, and years of experience, were not significant predictors in this model. The regression model explained 35% of the variance in LA (adjusted *R*^2^ = 0.35). Multicollinearity diagnostics revealed acceptable VIFs (all VIFs < 1.5). Model assumptions were checked, and appropriate transformations were applied to ensure robustness of the findings.

### 4.4. Enhanced Regression Model

The analysis required several empirical modifications to enhance its robustness and accuracy. It incorporated control variables such as leadership position, years of experience, educational level, and age to account for potential confounders that could influence the outcomes. In addition, the research introduced an interaction term between gender and EI variables to examine whether EI affects access to leadership roles differently for male and female nurses. The study also explored hospital-to-hospital variations to assess whether gender-related disparities in LA persist across different healthcare facilities. The findings of the regression model, which detail these relationships, are presented in Table 6.

#### 4.4.1. Interaction Effect: Gender × EI on LA

The interaction analysis reveals essential information about how leadership development differs between male and female nurses. While EI promotes LA for nurses, the effect is substantially weaker for female nurses according to the significant negative interaction coefficient (−0.1111). Emotionally intelligent female nurses still encounter institutional obstacles that impede their advancement to leadership positions, but their male counterparts with equivalent EI do not experience the same number of obstacles.

**Figure 4:**
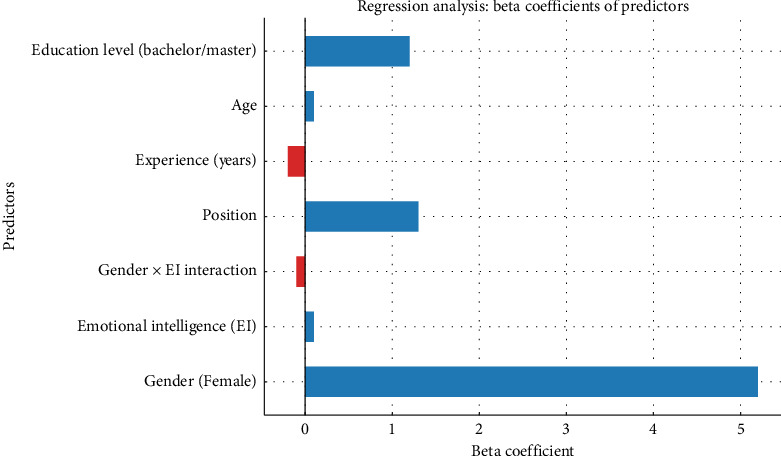
Beta coefficients of predictors.

The model's regression coefficients appear as bars in horizontal format. Each regression coefficient has a different color scheme, where blue indicates a positive value and red indicates a negative value (Figure 4).

**Figure 5:**
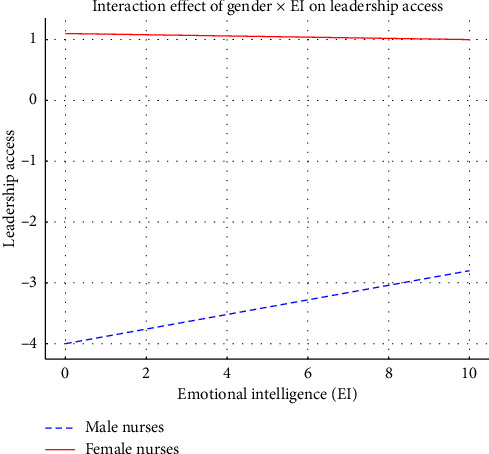
Interaction effect: emotional intelligence and leadership access by gender.

This graph shows how EI affects leadership opportunities differently between male and female nursing staff. The blue dashed line shows male nurses, while the solid red line illustrates female nurses, indicating that women gain less advantage from EI in terms of LA (Figure 5).

Nurses working in different hospital settings have different gender disparity levels because they are part of specific institutional environments. This study did not conduct a complete multilevel analysis but preliminary findings indicate that healthcare institutions might show different levels of leadership gender discrimination. Research must focus on hospital-based policies, workplace cultures, and leadership selection procedures because hospitals differ in their distribution of leadership positions between genders.

### 4.5. Comparative Analysis

In this section, descriptive statistics, independent samples *t*-test, and regression analysis were used to compare gender differences and EI on LA prediction among nurses.

The key findings from the descriptive statistics, *t*-test, and regression analysis for EI, LA, and CD opportunities are summarized in Table 7.

• Descriptive statistics (mean/SD): The mean and SD for male and female nurses on each variable are given in this column.
• *T*-test results (*t*-value and *p* value): Each variable has a *t*-value and *p* value, which tells us if there is a statistically significant difference between males and females.
• Regression results (beta and *p* value): This predictor's beta coefficient for EI and gender is the effect of the predictor on LA. CD was not included in the regression analysis.

**Figure 6:**
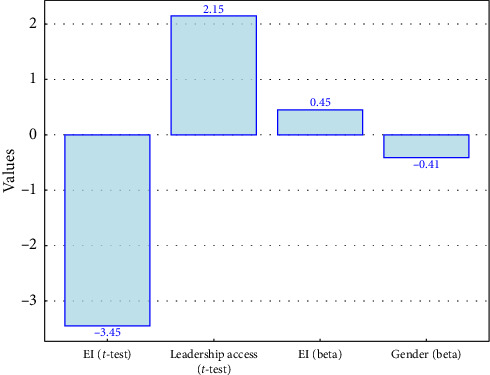
Comparison of *t*-values and beta coefficients for emotional intelligence and leadership access.

Figure 6 combines the results from the *t*-test and regression analysis, showing the t-values for gender differences and beta coefficients for the prediction of leadership access.

• EI (*t*-test): This bar represents the *t-*value for the difference in EI between male and female nurses.
• LA (*t*-test): This bar represents the *t*-value for the difference in LA between male and female nurses.
• EI (beta): This bar represents the beta coefficient for EI in predicting LA.
• Gender (beta): This bar represents the beta coefficient for gender in predicting LA.

## 5. Discussion

This research reveals significant gender differences in EI, LA, and CD among nurses in Jordanian healthcare facilities. Female nurses demonstrated notably higher EI scores compared to their male counterparts. However, despite this advantage, male nurses reported greater access to leadership opportunities. CD availability did not differ significantly between genders. These findings suggest that institutional factors may limit LA for female nurses despite their strengths in EI.

The regression analysis demonstrated that EI is a significant positive predictor of LA (*β* = 0.450, *t* = 3.65, *p* < 0.001), supporting the hypothesis that nurses with higher EI are more likely to obtain leadership positions. Gender also significantly influenced LA (*β* = −0.410, *t* = −2.20, *p*=0.030), with female nurses experiencing reduced access compared to males, even after accounting for EI levels. The interaction between gender and EI indicated that while EI enhances LA for both genders, this effect is less pronounced for female nurses (*β* = −0.1111, *t* = −5.814, *p* < 0.001). These findings suggest that although EI plays a crucial role in leadership selection, it does not fully account for the gender disparities observed in leadership access.

This research identifies gender differences in LA but does not establish deliberate organizational discrimination against women. The higher LA reported by male nurses cannot be interpreted as evidence that institutions intentionally exclude female nurses. While this study did not directly examine informal leadership networks, mentorship structures, or workplace culture, these factors may contribute to the observed disparities and warrant further investigation. Career advancement in nursing depends not only on formal policies but also on the strength of professional networks and workplace dynamics. To promote equitable leadership opportunities, hospital management should implement structured mentorship programs, transparent promotion criteria, and inclusive leadership development initiatives.

These findings suggest the need for institutional reforms that go beyond general policy statements. Hospitals and nursing leadership should consider implementing structured leadership development frameworks that ensure transparent selection criteria, routine performance reviews linked to advancement, and equal access to mentorship and sponsorship opportunities. Promotion pathways should be formalized through competency-based progression models, where leadership potential is assessed through objective tools rather than informal nomination or seniority alone. Regular audits of gender representation in supervisory and managerial roles may also help track progress and accountability.

Previous studies have established the existence of gender-related leadership disparities, and the present research extends these explanations by highlighting additional factors. Prezerakos [8] emphasized that while EI is a vital leadership quality, workplace systems and organizational standards often restrict female nurses from advancing into leadership roles. Complementing this, Deng et al. [11] found that male nurses benefit from stronger professional mentorship networks and sponsorship programs, which contribute to their enhanced leadership opportunities. In contrast, consistent with Gunawan and Aungsuroch [9], this study observed no significant gender differences in access to CD programs, suggesting that professional training and mentorship initiatives within Jordanian healthcare institutions are largely gender-neutral.

The study indicates that equal access to CD programs alone does not explain leadership disparities, as female nurses still face limited advancement despite comparable training. Individual factors, such as career aspirations, networking abilities, and leadership self-confidence, also influence outcomes. Healthcare institutions' perceptions and promotion decisions are shaped by cultural norms and unconscious biases regarding leadership styles. Stereotypes associating assertiveness and decisiveness with male leaders may subtly affect leadership selection, operating beyond formal policies and without overt discrimination.

The research faces various problems with its methodology. The quality of being cross-sectional in the study prevents researchers from making direct causal connections to explain why gender differences in leadership roles happen between workplace systems and parallel dynamics. The study would benefit from longitudinal analysis to reveal how LA develops throughout time. The research data consist of self-reported statements that could lead respondents to show positive bias towards aspects such as EI and career advancement plans. Additional research needs to include independent assessments that confirm leadership competence accurately through external evaluation. The study controlled for gender along with EI and CD possibilities but did not adjust for aspects such as mentorship quality or workplace cultural elements together with leadership selection standards that probably affect LA at work.

The study results apply only to the Jordanian healthcare system, because women represent 67% of nurses but hold less than 20% of leadership positions [2]. This regional pattern matches international trends but institutional rules together with cultural norms create barriers for direct comparison between Western and Asian healthcare settings. Future studies need to examine if gender-related inequalities exist across different healthcare facilities while focusing on countries that promote leadership advancement differently.

The research establishes quantitative evidence about gender inequalities in nurse leadership positions in Jordan without proving direct discrimination or explicit policy biases at work. Several combined factors including professional career reasons as well as mentorship availability and organizational work environment determine how leadership paths develop. EI serves as a strong indicator of leadership access, yet it fails to explain all the factors behind gender imbalances when accessing leadership positions. Future research needs to investigate informal leadership networks and cultural leadership style perceptions to develop specific interventions that will achieve gender equity in nursing leadership.

## 6. Significance of Our Work

Our study offers several significant contributions to the existing literature on gender disparities in nursing leadership. While previous studies have found gender imbalances in leadership across different healthcare sectors [25, 34], our research is the first to quantify the effect of EI and gender on LA in the Jordanian nursing sector. Using a regression model, we found that LA is negatively affected by gender, even controlling for EI, not previously considered in prior studies.

Our work also addresses the regional context of Jordan, which is frequently omitted from global literature on nursing leadership. Our findings are particularly important for healthcare policymakers in similar regions if the cultural and societal norms of Middle Eastern countries exacerbate the challenges faced by female nurses in accessing leadership roles. The result of this research stands in contrast to previous studies in Western healthcare systems [25] and adds to the call for targeted policy interventions to achieve gender equity in leadership for female nurses in Jordan. Recent evidence also highlights that empowering nurses through equitable leadership opportunities is essential for enhancing professional autonomy and facilitating the integration of emerging technologies, such as artificial intelligence, into nursing practice [35, 36].

### 6.1. Implications for Practice

The findings of this study have important implications for healthcare leadership and management. First, healthcare organizations should put leadership development programs for male and female nurses at the top of the priority list. Previous research has shown that leadership training programs can have a large impact on a nurse's ability to make the leap into leadership roles [9], but our findings indicate that female nurses are not represented in these leadership training programs currently.

Second, since leadership success is dependent on EI, as Vyas [26] and Prezerakos [8] show, healthcare organizations should include EI development in their leadership training programs. Doing so will enable them to aid male and female nurses in improving their leadership potential and developing their team management and decision-making potential.

Finally, our findings underline that policy interventions to eliminate gender bias in leadership selection are needed. Despite their high emotional intelligence, female nurses are perceived as having lower leadership ability than their male counterparts. In order to bridge the leadership gap and create a more inclusive healthcare, mentoring and leadership training should be performed, specifically for the female nurses, to help address these biases.

## 7. Conclusion

### 7.1. Implications for Practice and Policy

While female nurses have higher EI, a key leadership trait, this study reveals that they continue to have less access to leadership roles than their male counterparts. Although this study did not assess pay gaps directly, the findings suggest that structural barriers to career advancement persist. Addressing these disparities requires organizational policy reform, targeted leadership training programs, and a workplace culture that actively supports equitable progression. The LAS developed in this study may also serve as a useful tool for human resource departments to identify perceived leadership barriers and inform equity-oriented strategies. Recent studies suggest that broader institutional practices, such as green HR strategies, can also enhance organizational performance and nurse engagement [37].

However, given that EI is a strong predictor of access to leadership, hospitals and healthcare systems should advance work on the development of leadership programs to foster equitable career paths for nursing through leadership positions, regardless of gender. The leadership opportunities for gender inclusivity should be taken by healthcare policymakers to provide targeted leadership training and mentorship programs for female nurses.

Institutional reforms should aim to eliminate gender bias in employment and promotion and hiring people based on their competence and not according to the traditional gender norms, rather than improve women's participation in decision-making. Offering flexible PD programs, rather than being restricted by cultural expectations to remain in this role, flexible scheduling, online courses, and travel support programs can bridge this gap encouraging more flexible development programs.

### 7.2. Recommendations for Further Research

Future studies should incorporate qualitative insights into the experiences of female nurses regarding LA, going beyond statistical analysis. Cross-country comparisons could help determine whether gender disparities are universal or more pronounced in specific healthcare systems. In addition, future research should consider longitudinal follow-up to examine how LA and CD trajectories evolve over time. In addition, exploring intersectional factors, such as the combined effects of gender, age, and healthcare may offer a more nuanced understanding of disparities in leadership opportunities among nurses.

## Figures and Tables

**Table 1:** Cronbach's alpha and reliability scores.

Measurement instrument	Number of items	Sample size used	Cronbach's alpha
Emotional intelligence scale (EIS)	33	186	0.87
Leadership access scale (LAS)	10	186	0.85
Career development scale (CDS)	6	186	0.83

**Table 2:** Demographic and professional characteristics based on gender.

Variable	Category	Male (*n* = 85), *n* (%)	Female (*n* = 101), *n* (%)
Age	≤ 34 years	18 (21.2%)	25 (24.8%)
	35–44 years	46 (54.1%)	54 (53.5%)
	≥ 45 years	21 (24.7%)	22 (21.7%)

Educational level	Bachelor	63 (74.1%)	75 (74.3%)
	Master's	22 (25.9%)	26 (25.7%)

Years of experience	6–10 years	17 (20.0%)	19 (18.8%)
	11–15 years	37 (43.5%)	43 (42.6%)
	16–20 years	17 (20.0%)	21 (20.8%)
	≥ 21 years	14 (16.5%)	18 (17.8%)

Experience as a first-line manager	1–5 years	7 (8.2%)	8 (7.9%)
	6–10 years	32 (37.6%)	38 (37.7%)
	11–15 years	23 (27.1%)	28 (27.7%)
	≥ 16 years	23 (27.1%)	27 (26.7%)

Current position	Head nurse	14 (16.5%)	21 (20.8%)
	Charge nurse	33 (38.8%)	39 (38.6%)
	Acting head nurse	8 (9.4%)	10 (9.9%)
	Acting charge nurse	30 (35.3%)	31 (30.7%)

Type of hospital	Primary hospital	27 (31.8%)	36 (35.6%)
	Secondary hospital	16 (18.8%)	19 (18.8%)
	Tertiary hospital	42 (49.4%)	46 (45.6%)

Nursing specialty unit	Cardiology	4 (4.6%)	5 (5.0%)
	Critical care	20 (23.3%)	14 (13.8%)
	Emergency	12 (14.0%)	8 (7.9%)
	Geriatric care	9 (10.5%)	6 (5.8%)
	Medical	20 (23.3%)	11 (10.9%)
	Surgical	16 (18.5%)	9 (8.9%)
	Maternal care	0 (0.0%)	25 (24.8%)
	Pediatric care	0 (0.0%)	15 (14.9%)
	Peri-operative	3 (3.5%)	4 (4.0%)
	Operation theater	2 (2.3%)	4 (4.0%)

**Table 3:** Level of emotional intelligence (*N* = 186).

Categorization	Frequency (*n*)	Percentage (%)
Very high	40	21.5
High	42	22.6
Average	35	18.8
Low	36	19.4
Very low	33	17.7

**Table 4:** Descriptive statistics of participants (*N* = 186).

Variable	Mean ± SD	Median (IQR)	Min	Max
Emotional intelligence (EI)	81.7 ± 5.1	82 (78–85)	66	96
Leadership access (LA)	5.7 ± 2.0	6 (4–7)	1	10
Career development (CD)	5.9 ± 2.1	6 (4–7)	1	10
Professional development (PD)	5.2 ± 2.0	5 (3–7)	1	10

**Table 5:** Independent samples *t*-test results for gender differences.

Variable	Mean (male)	Mean (female)	*t*-value	*p* value
Emotional intelligence (EI)	78.5	84.2	−3.45	0.001
Leadership access	6.2	5.4	2.15	0.034
Career development	7.0	6.8	0.76	0.442

**Table 6:** Regression analysis of leadership access.

Predictor	Beta coefficient	*t*-value	*p* value
Intercept	−4.0351	−4.416	*p* < 0.001
Gender (female)	5.2258	4.873	*p* < 0.001
Emotional intelligence (EI)	0.0866	6.981	*p* < 0.001
Gender × EI interaction	−0.1111	−5.814	*p* < 0.001
Position	1.1907	3.563	*p* < 0.001
Experience (years)	−0.1701	−6.544	*p* < 0.001
Age	0.0857	5.218	*p* < 0.001
Education level (bachelor/master)	1.1907	3.563	*p* < 0.001

**Table 7:** Comparison of descriptive, *t*-test, and regression results.

Variable	Descriptive statistics		*T*-test results		Regression results	
	Mean (M/F)	SD (M/F)	*t*-value	*p* value	Beta	*p* value
Emotional intelligence (EI)	78.5/84.2	5.1/4.7	−3.45	0.001	0.450	0.001
Leadership access	6.2/5.4	1.8/2.1	2.15	0.034	−0.410	0.030
Career development	7.0/6.8	1.6/1.5	0.76	0.442	—	—

**Table 8:** LAS' item analysis and Cronbach's alpha.

Item number	Leadership access scale items	Cronbach's alpha if item deleted
LAS-1	I have access to mentorship programs that support my leadership development	0.82
LAS-2	I am provided with opportunities to attend leadership training programs	0.83
LAS-3	I am included in discussions about departmental policies and leadership decisions	0.81
LAS-4	I am encouraged to apply for leadership positions when they become available	0.84
LAS-5	My supervisors actively support my leadership development	0.82
LAS-6	I receive fair and transparent evaluations regarding leadership promotions	0.83
LAS-7	My workplace has structured pathways for leadership advancement	0.81
LAS-8	Leadership opportunities are equally available to male and female nurses in my institution	0.84
LAS-9	I feel encouraged to pursue leadership roles within my organization	0.82
LAS-10	Leadership potential is recognized based on competence rather than gender	0.83

**Table 9:** LAS' items.

Item number	Statement
LAS-1	I have access to mentorship programs that support my leadership development
LAS-2	I am provided with opportunities to attend leadership training programs
LAS-3	I am included in discussions about departmental policies and leadership decisions
LAS-4	I am encouraged to apply for leadership positions when they become available
LAS-5	My supervisors actively support my leadership development
LAS-6	I receive fair and transparent evaluations regarding leadership promotions
LAS-7	My workplace has structured pathways for leadership advancement
LAS-8	Leadership opportunities are equally available to male and female nurses in my institution
LAS-9	I feel encouraged to pursue leadership roles within my organization
LAS-10	Leadership potential is recognized based on competence rather than gender

## Data Availability

The data that support the findings of this study are available on request from the corresponding author. The data are not publicly available due to privacy or ethical restrictions.

## Appendix A: LAS and Development and Validation of the LAS

The LAS functions as an instrument specifically designed to evaluate nurses' perception of their access to leadership opportunities through mentorship programs, training initiatives, managerial positions, and institutional support for career advancement. The instrument was developed to evaluate LA in nursing because the Jordanian healthcare sector lacked an appropriate validated scale.

The development process followed a multistep approach:

Literature review: identification of key dimensions related to LA in nursing.

Expert consultation: review and refinement of the scale by a panel of five healthcare leadership experts.

Pilot testing: pretesting with 30 nurses to assess item clarity and cultural relevance.

Reliability and validity testing: analysis of internal consistency using Cronbach's alpha, item-total correlations, and factor analysis.

The final version of the LAS consists of 10 items, each rated on a 5-point Likert scale (1 = strongly disagree and 5 = strongly agree). The scale demonstrated high internal consistency, with an overall Cronbach's alpha of 0.85, indicating strong reliability.

## A.1. Scale Items and Reliability Analysis

Table A1 presents the final 10 items of the LAS along with their Cronbach's alpha if the item was deleted, providing insight into the internal consistency of each item.

None of the individual items significantly decreased the overall reliability of the scale, confirming the robustness of the instrument.

## Appendix B: LAS

Table A2 displays the LAS. Participants are asked to rate the extent of their agreement with each statement about LA in their workplace, using the following five-point scale:

1 = strongly disagree, 2 = disagree, 3 = neutral, 4 = agree, and 5 = strongly agree.

## C.1. Development Process

**Item generation:**

Items were initially derived from a comprehensive literature review on LA in nursing [8, 9].

Multiple LA barriers such as mentorship access and training availability and decision-making participation and institutional policies were recognized.

**Expert review:**

A group of five healthcare management and nursing leadership experts evaluated the preliminary items.

The panel modified the wording to enhance clarity and ensure that the items would be relevant to Jordanian healthcare organizations.

**Pilot testing:**

The preliminary version of the scale underwent testing with 30 nurses distributed among three different hospital settings, which included public and private as well as military hospitals.

The participants assessed the clarity of items as well as the cultural appropriateness and readability of the scale.

**Reliability and validity testing:**

The first pilot analysis showed Cronbach's alpha reliability at 0.83, but it rose to 0.85 after making small adjustments to the instrument.

All scale items passed tests, showing their meaningful contribution to the construct assessment.

The ten scale items demonstrated convergence through factor analysis onto one main factor, thus validating the unique single-dimensionality of the measure.

The LAS offers nurses a reliable tool to measure their LA effectively. The instrument provides important institutional data about leadership advancement barriers in nursing through its assessment of mentorship access and training possibilities and decision-making involvement and gender equality. Research employing the LAS should continue by validating its cross-cultural application while investigating leadership inequality patterns across multiple healthcare facilities.
